# Indicators of central sensitization in chronic neuropathic pain after spinal cord injury

**DOI:** 10.1002/ejp.2028

**Published:** 2022-09-05

**Authors:** Robin Lütolf, Jan Rosner, Armin Curt, Michèle Hubli

**Affiliations:** ^1^ Spinal Cord Injury Center Balgrist University Hospital, University of Zurich Zurich Switzerland; ^2^ Department of Neurology University Hospital Bern, Inselspital, University of Bern Bern Switzerland

## Abstract

**Background:**

Central sensitization is considered a key mechanism underlying neuropathic pain (NP) after spinal cord injury (SCI).

**Methods:**

Two novel proxies for central sensitization were investigated in thoracic SCI subjects with (SCI‐NP) and without NP (SCI‐nonNP) compared to healthy controls (HC). Specifically, temporal summation of pain (TSP) was investigated by examining pain ratings during a 2‐min tonic heat application to the volar forearm. Additionally, palmar heat‐induced sympathetic skin responses (SSR) were recorded in order to reveal changes in pain‐autonomic interaction above the lesion level. Pain extent was assessed as the percentage of the body area and the number of body regions being affected by NP.

**Results:**

Enhanced TSP was observed in SCI‐NP (+66%) compared to SCI‐nonNP (−75%, *p* = 0.009) and HC (−59%, *p* = 0.021). In contrast, no group differences were found (*p* = 0.685) for SSR habituation. However, pain extent in SCI‐NP was positively correlated with deficient SSR habituation (body area: *r* = 0.561, *p* = 0.024; body regions: *r* = 0.564, *p* = 0.023).

**Conclusions:**

These results support the value of TSP and heat‐induced SSRs as proxies for central sensitization in widespread neuropathic pain syndromes after SCI. Measures of pain‐autonomic interaction emerged as a promising tool for the objective investigation of sensitized neuronal states in chronic pain conditions.

**Significance:**

We present two surrogate readouts for central sensitization in neuropathic pain following SCI. On the one hand, temporal summation of tonic heat pain is enhanced in subjects with neuropathic pain. On the other hand, pain‐autonomic interaction reveals potential advanced measures in chronic pain, as subjects with a high extent of neuropathic pain showed diminished habituation of pain‐induced sympathetic measures. A possible implication for clinical practice is constituted by an improved assessment of neuronal hyperexcitability potentially enabling mechanism‐based treatment.

## 1 INTRODUCTION

Neuropathic pain (NP) is a common complication after spinal cord injury (SCI) (Burke et al., 2017; Warner et al., 2019). The development and maintenance of NP are linked to neuronal hyperexcitability as well as disinhibition due to a malfunctioning endogenous pain modulation (Baron et al., 2010; Baron et al., 2013; Defrin et al., 2022). The shifted balance from anti‐ to pro‐nociceptive processes, manifested in increased responsiveness of nociceptive neurons, i.e. central sensitization, presumably underlies various pain syndromes (Gruener et al., 2016; Kutch et al., 2017; Staud et al., 2014; Willett et al., 2020; Yarnitsky et al., 2014; Zanette et al., 2010). The hyperexcitable state of the nociceptive neuraxis has been assessed with different measures (Arendt‐Nielsen et al., 2018), including enhanced temporal summation of pain (TSP) (Curatolo et al., 2001; Price et al., 2002; Staud et al., 2001; Staud et al., 2008) and loss of habituation to noxious stimuli (de Tommaso et al., 2011; Kumru et al., 2012; Olesen et al., 2013) in a variety of chronic pain cohorts. According to the concept postulated by Arendt‐Nielsen et al. (2018), patients with central sensitization are not only characterized by changes in gain‐of‐function and reduced pain thresholds at painful but also in remote non‐painful body areas. With regard to SCI this concept translates to the necessity of testing in sensory intact body areas above the level of injury.

In SCI, the assessment of TSP has been performed using repetitive phasic (Defrin et al., 2001; Eide et al., 1996; Konopka et al., 2012) and tonic (Albu et al., 2015; Gruener et al., 2016; Scheuren et al., 2019) noxious stimuli. Generally, studies found a higher occurrence and magnitude of TSP in subjects with SCI with NP (SCI‐NP) compared to those without (SCI‐nonNP) and healthy controls (HC) (Defrin et al., 2001; Gruener et al., 2016). The mechanisms underlying chronic pain have been suggested to be more closely related to dynamic changes in pain perception during tonic stimuli compared to static pain sensitivity, e.g. pain thresholds (Kleinbohl et al., 1999). During the course of heat application, multiple peripheral and central neuronal mechanisms are activated with partial temporal overlap (Price & Dubner, 1977; Tousignant‐Laflamme et al., 2008). Common denominators are an initial decrease of pain rating, i.e. adaptation (Gruener et al., 2016; Scheuren et al., 2019), followed by a subsequent increase, i.e. TSP, the latter potentially reflecting spinal sensitization, i.e. long‐term potentiation of dorsal horn neurons at the corresponding spinal segment (Potvin et al., 2008; Tousignant‐Laflamme et al., 2008). Whilst previous protocols were not specifically designed to track the full temporal spectrum and amplitude of pain modulatory processes (Albu et al., 2015; Gruener et al., 2016), a long (2 min) heat stimulus was used in order to reveal such a spectrum from a peripheral adaptation to a subsequent summation of the stimulated nociceptive neurons (Granot et al., 2006; Tousignant‐Laflamme et al., 2008). We aimed to investigate changes in pain perception during prolonged heat application in subjects with SCI and to explore the relation to NP characteristics.

The second sign of central sensitization, i.e. deficient habituation to noxious stimuli, has been reported in various chronic pain conditions by employing pain ratings (Albu et al., 2015; de Tommaso et al., 2005; de Tommaso et al., 2011; Kumru et al., 2012; Smith et al., 2008) and pain‐related evoked potentials (Albu et al., 2015; de Tommaso et al., 2005; de Tommaso et al., 2011; de Tommaso et al., 2017; Hullemann et al., 2017; Kumru et al., 2012; Valeriani et al., 2003; Vossen et al., 2015). In SCI‐NP, deficient habituation of contact heat‐evoked potentials after stimulation above the lesion level has been shown compared to SCI‐nonNP and HC, implying either or both pro‐ (increased excitability in pain processing areas) and anti‐nociceptive processes (decreased activity in centres regulating habituation) (Kumru et al., 2012). Besides pain ratings and evoked potentials, the nociceptive system can be investigated by employing objective and simple measures of its interaction with the autonomic nervous system (Benarroch, 2006), e.g. pain‐induced sympathetic skin responses (SSR). Here, autonomic responses to noxious stimulation were leveraged in order to reveal increased responsiveness in the nociceptive system. Altered autonomic function per se was not considered to be related to the pathophysiology of NP, but employing SSRs merely captures enhanced pain‐autonomic coupling as an objective measure of pain processing. As changes in general neuronal excitability have been tracked above the lesion using neuroimaging, neurophysiology and animal models (Gruener et al., 2016; Kutch et al., 2017; Staud et al., 2014; Willett et al., 2020; Yarnitsky et al., 2014; Zanette et al., 2010), we sought to provide additional lines of evidence from pain‐autonomic interactions. In that regard, the amplification of SSR has recently been discussed as a surrogate marker of experimentally‐induced central sensitization in healthy controls (HC) (Scheuren et al., 2020). Additionally, this measure of pain‐autonomic interaction was investigated in fibromyalgia (de Tommaso et al., 2017), migraine (Ozkul & Ay, 2007) and central pain in Parkinson's disease (Schestatsky et al., 2007) revealing hyperexcitability within the central nervous system. Therefore, our second aim was to explore SSR habituation as another sign of increased general neuronal excitability in SCI‐NP. We hypothesized that subjects with SCI suffering from below‐level NP will display signs of central sensitization, i.e. enhanced TSP and diminished SSR habituation, that can be discerned above the level of the lesion.

## 2 METHODS

### 2.1 Subjects

This study was carried out in a total of 48 subjects. The three groups included subjects with chronic SCI, either with NP (*n* = 20) or without NP (*n* = 14), as well as HC (*n* = 14). All three groups were age‐ and sex‐matched, whilst the SCI cohorts were also matched in lesion level, severity and time since injury. SCI inclusion criteria comprised an SCI for at least 1 year and a neurological level of injury between T1 and T12. The reasons for including only these levels of the lesion were twofold: (i) fully intact sensory function in dermatomes up to and including T1 in order to assess the volar forearm as a sensory intact area above the neurological level of injury and (ii) no lumbar or sacral lesions in order to exclude potential peripheral lesions and their contribution to the neuropathic pain. Exclusion criteria for the SCI cohort were neurological disorders other than SCI such as multiple sclerosis and Parkinson's disease, psychiatric or cognitive conditions interfering with the study, and pregnancy. Exclusion criteria for the HC comprised of pregnancy, any history or signs of a neurological condition, any history of a psychiatric condition and acute or chronic pain condition, as well as chronic medication intake (except contraceptives).

### 2.2 Study design

The study was designed as a one‐visit, cross‐sectional study with recruitment from October 2017 to November 2018. All subjects provided written informed consent, and all procedures were in accordance with the Declaration of Helsinki. The study was approved by the local ethics board ‘Kantonale Ethikkommission Zürich, KEK’ (ref.number: EK‐04/2006, PB_2016–02051, cinicaltrial.gov: NCT02138344). The visit started with subjects filling out the German version of the Pain Catastrophizing Scale (PCS) (Sullivan et al., 1995) and the Beck's Depression Inventory‐II (BDI‐II) (Robinson & Kelley, 1996) in order to assess the possible confounding effects of pain catastrophizing and mood on pain experience (Sullivan et al., 2001). The following clinical assessment, pain phenotyping and testing of pain‐autonomic interaction were done in a quiet room with an ambient temperature of 21–24.5°C.

### 2.3 Clinical assessment and pain phenotyping

Subjects with SCI underwent a standard clinical examination according to the International Standards for Neurological Classification of SCI (ISNCSCI) by a trained neurologist to evaluate the neurological level of injury (NLI) as well as injury severity (American Spinal Injury Association Impairment Scale, AIS) (Kirshblum et al., 2020). The integrity of thermo‐nociceptive afferents at the testing site of the volar forearm was assessed with pinprick testing and cold as well as warm detection thresholds (CDT, WDT). The thermal thresholds were performed using a method of limits according to the recommendations of the German Research Network on Neuropathic Pain (Rolke et al., 2006). A contact heat stimulator (PATHWAY Pain & Sensory Evaluation System, Medoc Ltd., Ramat Yishai, Israel) with an advanced thermal stimulator (ATS) thermode (30 x 30 mm contact surface) was used. In brief, the average of three thresholds was taken, starting at a baseline temperature of 32°C (rate: 1°C/s, safety cut‐offs: 0°C and 50°C, ISI: 4–6 s). The pain phenotyping included a detailed assessment according to the guidelines of the International Spinal Cord Injury Pain (ISCIP) Classification group (Widerstrom‐Noga et al., 2014; Widerstrom‐Noga et al., 2016). The presence of cold allodynia was tested with a 25°C thermoroller (Somedic, Hörby, Sweden) and the presence of mechanical allodynia was tested with a brush and the International Spinal Cord Injury Data Set (ISCPDS) questionnaire (Widerstrom‐Noga et al., 2014). The quantification of NP was performed by pain drawings on two papers with standardized body charts (frontal and dorsal view) (Rosner et al., 2021). The perceived NP at the very moment was marked by the SCI‐NP group and further characterized by verbal descriptors (hot, burning, shooting, piercing, stinging, stabbing, sharp, throbbing, cramping) and pain intensity (numeric rating scale, NRS; ‘0’ = no pain, ‘10’ = worst pain imaginable). Subjects with a current neuropathic pain intensity of NRS ≥3 were assigned to the SCI‐NP group in order to examine moderate to severe NP syndromes (Hanley et al., 2008). The experimenter manually outlined the borders of the pain areas on the pain drawings. After digitalization, the sum of pixel count per pain drawing (frontal and dorsal) was analysed for the NP extent using an image analysis software (Inkscape version 0.48) (Rosner et al., 2021). Based on previous literature, pain extent was reported as the number of body regions (out of a total of 13: foot, shin, hip/thigh, buttocks, hand, forearm, arm, shoulder, stomach, chest, lower back, upper back and neck, with the first eight regions counted twice if affected bilaterally) being affected by NP (Gruener et al., 2016; Widerstrom‐Noga et al., 2008), and as the percentage of the total body area.

### 2.4 Tonic heat application

Tonic contact heat was applied at the non‐dominant volar forearm (above the level of injury) in an area with intact sensory function in order to use the dominant hand for computerized pain rating. The contact heat stimulator was used with the same ATS thermode as described above. Subjects were seated upright and placed the dorsal forearm of their non‐dominant side on a table. The tonic heat protocol comprised of a temperature increase from 32 to 45°C (heat‐pain coupling, 1°C/s) and a 2‐min constant heat application (Jutzeler et al., 2019). The destination temperature of 45°C could not have been exceeded due to the technical safety limits of the device (Sirucek et al., 2020). Subjects were blinded to the nominal temperature and were told that the thermode temperature could rise, remain stable or decrease. Subjects were familiarized on the dominant side and were instructed to continuously rate the perceived pain on a computerized visual analogue scale (CoVAS, left edge = no pain = 0; right edge = worst pain imaginable = 100) during the 2‐min testing period.

### 2.5 Pain‐autonomic interaction

Subjects were placed in a supine position and contact heat stimuli were applied with the same stimulator as described above, however, using the contact heat‐evoked potential stimulator (CHEPS) thermode. For the phasic contact heat stimuli, the baseline and destination temperature were set to 42 and 52°C, respectively (Haefeli et al., 2014; Jutzeler et al., 2016; Rosner et al., 2018). If the stimulation was not tolerated, the baseline temperature was reduced to 35°C. After a familiarization with the heat stimuli on the left volar forearm, 10 stimuli were applied to the right volar forearm, i.e. a sensory intact area above the level of lesion, in order to minimize the carry‐over effects from the tonic heat application. The inter‐stimulus interval ranged from 15 to 19 s, and the thermode positioning was slightly changed after each stimulus to avoid peripheral receptor fatigue (Greffrath et al., 2007). Subjects were instructed to rate the perceived pain of each heat stimulus on the NRS cued by an auditory signal provided 9 s after the heat stimulus. For the assessment of the habituation of pain‐autonomic interaction, the sympathetic sudomotor activity elicited by the contact heat stimuli was assessed. Ten SSRs were recorded with self‐adhesive recording electrodes (AMBU BlueSensor NF‐50‐K/W, Ambu, Denmark) attached to the palm of the left hand (contralateral to the applied heat stimulus) referenced to its dorsum. The skin was prepared with abrasive sandpaper and alcohol before placing the electrodes in order to reduce skin impedance. The SSR recordings were triggered to the heat stimuli and acquired using a customized LabVIEW program (V2.04 CHEP, ALEA Solutions, Zurich, Switzerland) with a recording time of 10 s including a one‐second pre‐trigger window. The signals were acquired at 2000 Hz using a preamplifier (20,000×, ALEA Solutions, Zurich, Switzerland) and processed with a moving average filter of 50 samples.

### 2.6 Data analysis and statistics

**FIGURE 1:**
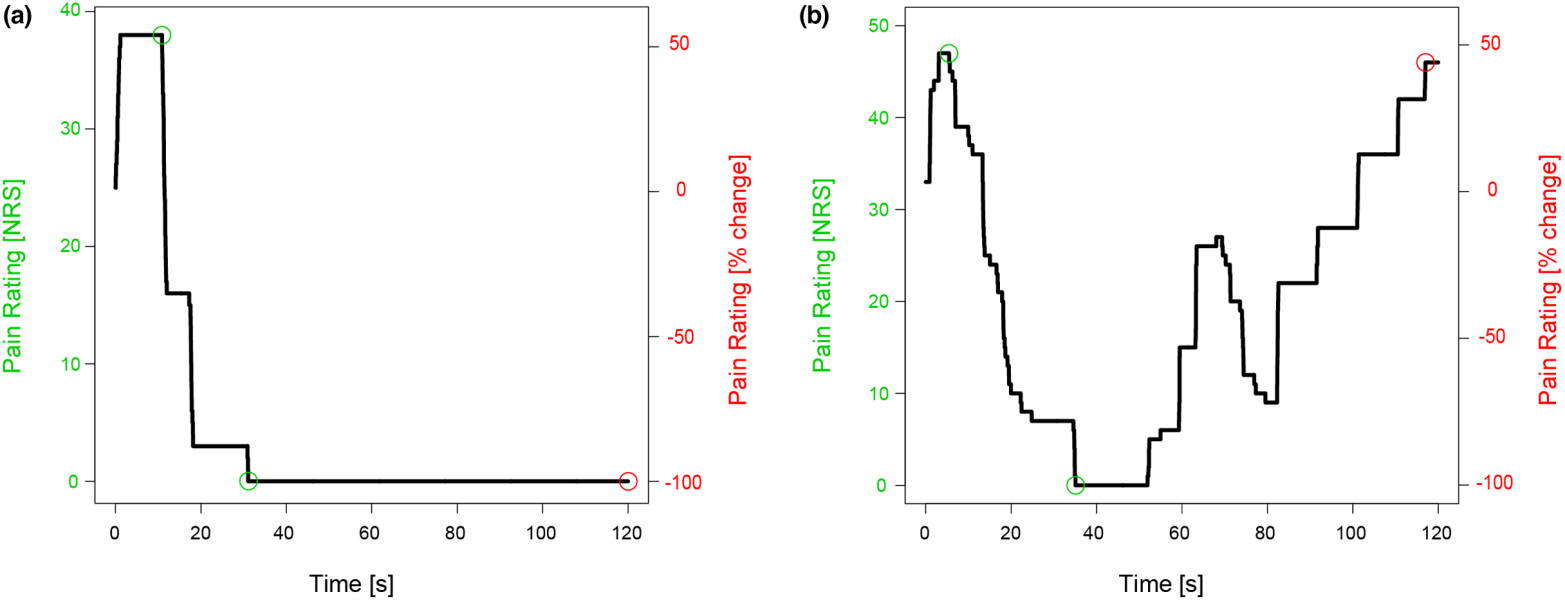
Representative examples of pain ratings during tonic heat application for two healthy controls. (a) Adaptation magnitude and with −100% temporal summation of pain (TSP). (b) Adaptation magnitude with +43.8% TSP magnitude. Markers are set for the start and end of adaptation (green) as well as for the resulting temporal summation of pain (red). The tonic heat profiles are shown on a numerical rating scale (NRS, left) as well as normalized to the rating at the ramp (right axis). Negative values illustrate lower pain ratings and positive values illustrate higher pain ratings compared to the ramp.

Tonic heat pain ratings could only be analysed if the stimulus was perceived as painful either at the beginning and/or the end of the 2‐min heat application. The pain ratings were normalized to the initial pain rating at the end of the heating ramp (when reaching 45°C) and plotted over the 120 s of heat pain stimulation. The magnitude of adaptation and TSP were calculated as follows: The adaptation magnitude was defined as the percentage difference between the peak pain rating after reaching the pre‐set stimulation temperature and the lowest pain rating throughout the 2‐min tonic heat application. The TSP magnitude was defined as the percentage difference between the rating at the end of the heating ramp and the final pain rating of the 2‐min tonic heat application (last minus first) (Erpelding & Davis, 2013; Pedersen et al., 1998). Negative values represent decreases in pain ratings throughout the tonic heat application, whereas positive values were assumed to reveal pro‐nociceptive mechanisms taking place during the 2‐min heat pain application. Representative examples in Figure 1 depict pain ratings and the detection of its phases during the tonic heat application, e.g. adaptation without the occurrence of TSP (Figure 1a), or adaptation and TSP (Figure 1b).

Regarding the SSR recordings, two examiners inspected the individual trials for artefacts, e.g. movement and coughing. Quantitative analysis of the SSR trials took into account the latency and the peak‐to‐peak amplitude. We used a customized algorithm using the R computing environment (R Studio version 4.0.4), for the quantitative analysis of the SSR trials. The SSR latency and amplitude were calculated on a single trial level. SSR latency was manually detected and the amplitude was automatically defined as the peak‐to‐peak of every single response. Each SSR signal underwent a final inspection to ensure that the latencies and amplitudes were correctly set. SSR latencies were considered pathological when exceeding two standard deviations of the mean latency of HC (de Tommaso et al., 2017). For SSR habituation, the amplitudes of single trials were calculated and further analysed as the mean of the last three (8th–10th stimulation) normalized to the first three SSR amplitudes for the habituation value in per cent. Here, pronounced habituation would result in negative values reflecting anti‐nociceptive mechanisms, whereas deficient habituation would result in positive values reflecting pro‐nociceptive mechanisms.

Statistical analyses were performed using RStudio (version 4.0.4). The normal distribution of the data was tested using the Shapiro–Wilk test and histograms. General linear mixed models (LMM, ‘lme’ function from R package ‘nlme’, with subjects as a random effect) were used to assess the main effect of group (SCI‐NP, SCI‐nonNP and HC) on the following dependent variables (one model each): Age, time since injury, PCS score, BDI‐II score, CDT, WDT, pain rating at the ramp, adaptation magnitude, TSP magnitude, contact heat pain ratings, habituation of contact heat pain ratings, SSR amplitude, habituation of SSR amplitude and SSR latency. Regarding the temporal aspect of pain ratings during the tonic heat application, the main effect of time points (0, 30, 60, 90, 120 s) on adaptation and TSP magnitude was also assessed. The interaction effect (group × time point, using Wald test) was first included in the model and removed afterwards if it was not significant. Models on adaptation and TSP magnitude were adjusted for the pain rating at the ramp. Additionally, the main effect of allodynia (subjects with SCI with or without allodynia) was assessed on TSP magnitude as well as SSR habituation. Inspection of model residuals showed that requirements for LMM were met for all parameters.

Further, Pearson correlations were performed to investigate the association of SCI‐NP characteristics, i.e. intensity and extent of SCI‐NP, as well as PCS and BDI‐II scores with TSP magnitude and SSR habituation.

## 3 RESULTS

### 3.1 Subjects

Two subjects had to be excluded from the analysis, resulting in a final sample of 46 subjects (SCI‐NP: *n* = 18, SCI‐nonNP: *n* = 14, HC: *n* = 14). The reasons for the exclusions were clinical evidence of a concomitant polyneuropathic syndrome and an inability to follow the experimental protocol due to strong medication side effects (both from the SCI‐NP group). The current pain medication included anti‐epileptic drugs (*n* = 9), non‐steroidal anti‐inflammatory drugs (*n* = 8), antidepressants (*n* = 4) and cannabinoids (*n* = 2). Demographics of all subjects and clinical characteristics of the SCI cohort are listed in Table 1. No difference in age was found between the three groups (*p* = 0.431) and time since injury between the two SCI groups (*p* = 0.919). However, significant group differences were found for PCS (*p* = 0.018) and BDI scores (*p* < 0.001). Post‐hoc testing revealed higher PCS and BDI scores for SCI‐NP (*p* = 0.025, *p* < 0.001, respectively) and SCI‐nonNP (*p* = 0.045, *p* = 0.028, respectively) compared to HC, but not between SCI‐NP and SCI‐nonNP (*p* = 0.994, *p* = 0.270, respectively). Importantly, pinprick scores tested at the volar forearm were normal in all subjects and no significant difference was observed for CDT (*p* = 0.147, NP: 30.5 ± 0.6°C; SCI‐nonNP: 30.3 ± 1.0°C; HC: 30.9 ± 0.8°C) and WDT (*p* = 0.107, NP: 36.5 ± 3.0°C; SCI‐nonNP: 35.3 ± 1.3°C; HC: 35.1 ± 1.2°C).

**TABLE 1:** Subject characteristics

	SCI‐NP	SCI‐nonNP	HC
Gender [f/m]	3/15	2/12	2/12
Age [year]	58.6 ± 9.0	53.9 ± 12.0	58.1 ± 11.0
PCS score [*p*]	11.1 ± 9.6*	10.8 ± 9.0*	3.3 ± 3.8
BDI‐II score [*p*]	8.1 ± 5.3**	5.7 ± 4.6*	1.4 ± 1.6
Time since injury [year]	18.1 ± 10.4	17.6 ± 13.1	–
AIS	9 A, 3 C, 6 D	7 A, 2 C, 5 D	–
NLI	Th1‐12	Th4‐12	–
Aetiology [traumatic/nontraumatic]	12/6	11/3	–
Pain intensity [NRS]	5.6 ± 2.2	–	–
Pain extent [% of body area]	18.2 ± 16.0	–	–
Pain extent [# of body regions]	5.3 ± 2.0	–	–
Allodynia [yes/no]	10 / 8	–	–

*Note*: Demographics, SCI and neuropathic pain characteristics. Significance levels are reported for the comparison of the SCI groups with HC as * for *p* < 0.05, ** for *p* < 0.001.

Abbreviations: AIS, American spinal injury association impairment scale; AIS a, sensorimotor complete SCI; AIS C & D, sensorimotor incomplete SCI; BDI, Beck's depression inventory; HC, healthy controls; PCS, pain catastrophizing scale; SCI; spinal cord injury; SCI‐nonNP, SCI subjects without neuropathic pain; SCI‐NP, SCI subjects with neuropathic pain.

### 3.2 Temporal summation of tonic heat pain

**FIGURE 2:**
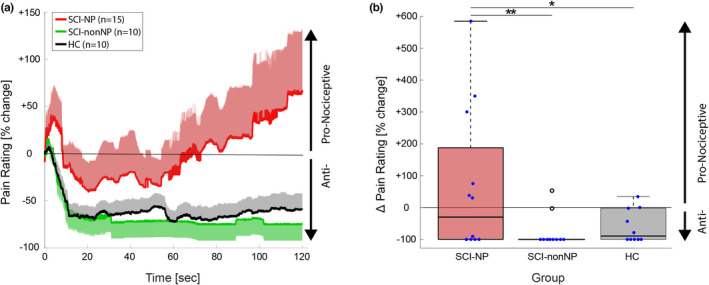
Pain ratings and temporal summation of pain (TSP) during tonic heat application. (a) Grand averages of tonic heat pain ratings in the three groups (SCI‐NP in red, SCI‐nonNP in green and HC in black). The grand averages are shown normalized to the rating at the ramp, and are plotted as the mean and standard error of the mean. (b) Quantification of the TSP magnitude for the three groups. Negative values illustrate lower pain ratings and positive values illustrate higher pain ratings compared to the ramp. HC, healthy controls; SCI‐nonNP, SCI subjects without neuropathic pain; SCI‐NP, SCI subjects with neuropathic pain; TSP, temporal summation of pain; VAS, visual analogue scale.

In total 35 tonic heat pain profiles (15 SCI‐NP, 10 SCI‐nonNP and 10 HC) were analysed, whilst 11 subjects (3 SCI‐NP, 4 SCI‐nonNP and 4 HC) had to be excluded as the heat stimulus did not elicit any painful percept which could be modulated over the course of the paradigm. Pain ratings at ramp showed a significant group difference (*p* = 0.047) with lower ratings for SCI‐NP (8.5 ± 12.8 VAS) compared to HC (23.0 ± 18.3 VAS, *p* = 0.040) but not to SCI‐nonNP (12.0 ± 9.9 VAS, *p* = 0.809 and *p* = 0.196, respectively). As displayed in Figure 2, all three groups showed a pronounced adaptation, but no significant difference was observed between the three groups (*p* = 0.570, SCI‐NP: −84.8 ± 29.2%, SCI‐nonNP: −93.1 ± 20.0%, HC: −94.7 ± 15.0%). The interaction of group and time point (*p* = 0.014) on TSP magnitude was only significant at 120 s, whereas the analysis at 30, 60 and 90 s revealed no difference between groups. Post‐hoc testing highlighted higher TSP magnitude at 120 s in SCI‐NP compared to SCI‐nonNP (+66% vs. −75%, *p* = 0.009) and HC (+66% vs. −59%, *p* = 0.021), but not between SCI‐nonNP and HC (−75% vs. −59%, *p* = 0.702). Interestingly, more than 50% of SCI‐NP subjects showed a TSP overpowering the ADT magnitude whilst this was only seen for 10% of SCI‐nonNP subjects and 10% of HC. Further analyses revealed no correlation of TSP magnitude with extent (*r* = −0.0812, *p* = 0.802 for percent of body area and *r* = 0.241, *p* = 0.451 for body regions) and intensity (*r* = 0.483, *p* = 0.112) of spontaneous NP. Interestingly, the PCS score positively correlated with the TSP magnitude in SCI‐NP (*r* = 0.620, *p* = 0.031), whereas the BDI score showed no correlation (*r* = −0.112, *p* = 0.744). Further, no difference in TSP was found between subjects with and without allodynia at or below the lesion level (*p* = 0.220).

### 3.3 Habituation of sympathetic skin responses

**FIGURE 3:**
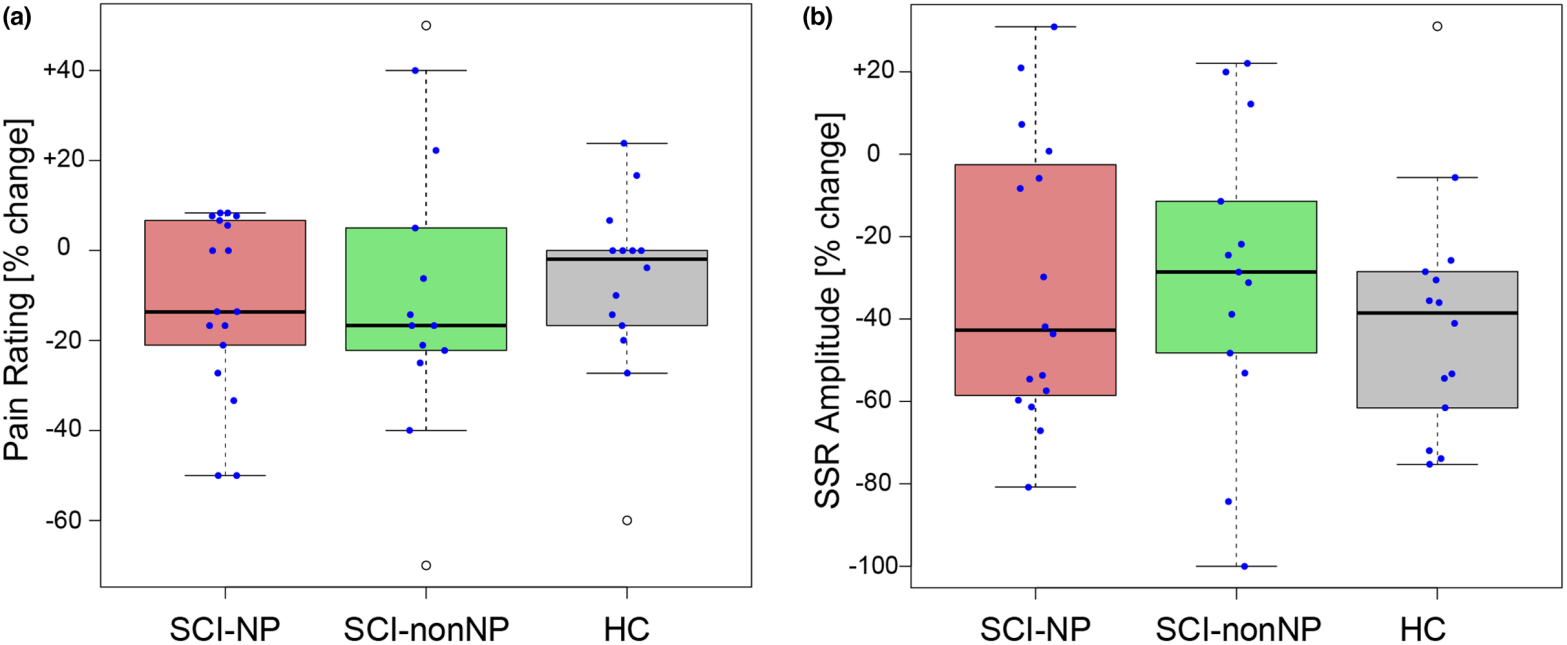
Habituation of contact heat pain ratings and SSR (sympathetic skin response) in the three groups. (a) no group difference was found with regard to pain rating habituation. (b) Habituation of SSR amplitude also revealed no group difference. HC, healthy controls; SCI‐nonNP, neuropathic pain‐free subjects; SCI‐NP, neuropathic pain subjects.

The SSR analysis was based on data from 44 out of the 46 subjects. One subject from the SCI‐NP group and one from the SCI‐nonNP group had to be excluded due to a technical problem and intolerable pain during phasic heat stimulation, respectively. In one SCI‐NP subject, the less intense stimulation protocol had to be used. No significant difference between the three groups was found for averaged pain ratings (SCI‐NP: 4.6 ± 2.0 NRS, SCI‐nonNP: 4.1 ± 1.8 NRS, HC: 4.8 ± 2.2 NRS, *p* = 0.590), habituation of pain ratings (SCI‐NP: −11.6 ± 19.8%, SCI‐nonNP: −8.8 ± 32.2%, HC: −7.5 ± 20.5%, *p* = 0.888), SSR amplitudes (SCI‐NP: 2326 ± 2220 μV, SCI‐nonNP: 2566 ± 1692 μV, HC: 3129 ± 1628 μV, *p* = 0.497) and SSR habituation (SCI‐NP: −31.5 ± 34.4%, SCI‐nonNP: −29.8 ± 36.8%, HC: −40.2 ± 29.0%, *p* = 0.685). Habituation of pain ratings and SSR for the three groups can be seen in Figure 3. There was a significant group difference in SSR latency (*p* = 0.005) with prolonged latencies seen in both SCI groups (SCI‐NP: 1.74 ± 0.15 s; SCI‐nonNP: 1.70 ± 0.18 s) compared to the HC (1.57 ± 0.07 s). Pathological SSR latencies were found in 14/30 subjects with SCI (7 SCI‐NP and 7 SCI‐nonNP).

**FIGURE 4:**
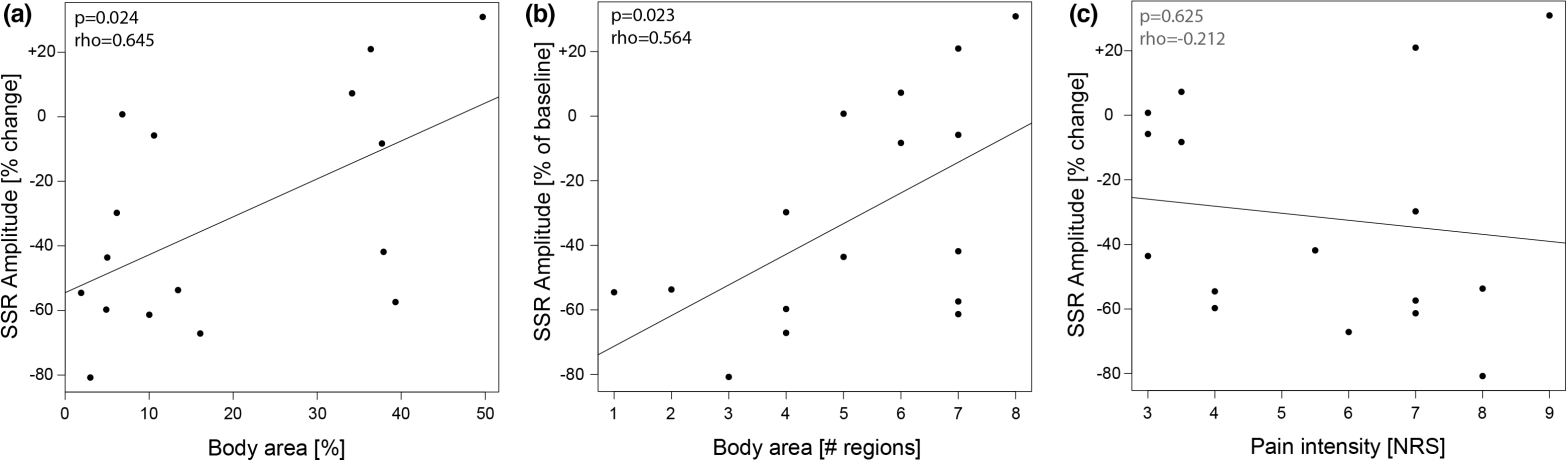
Correlation of SSR (sympathetic skin response) habituation with neuropathic pain characteristics. (a) Positive correlation of SSR habituation with body area affected by NP. (b) Positive correlation of SSR habituation with a number of body regions affected by NP. (c) no significant correlation of SSR habituation with NP intensity. NP, neuropathic pain; NRS, numeric rating scale.

Correlations of SSR habituation and spontaneous NP characteristics, i.e. intensity and spatial extent, are shown in Figure 4. Strikingly, SSR habituation correlated with NP extent (body area: *p* = 0.024, *r* = 0.561; body regions: *p* = 0.023, *r* = 0.564), but not with NP intensity (*p* = 0.625, *r* = −0.132). This highlights that the higher the NP extent, the lower the habituation of the SSR amplitude.

**FIGURE 5:**
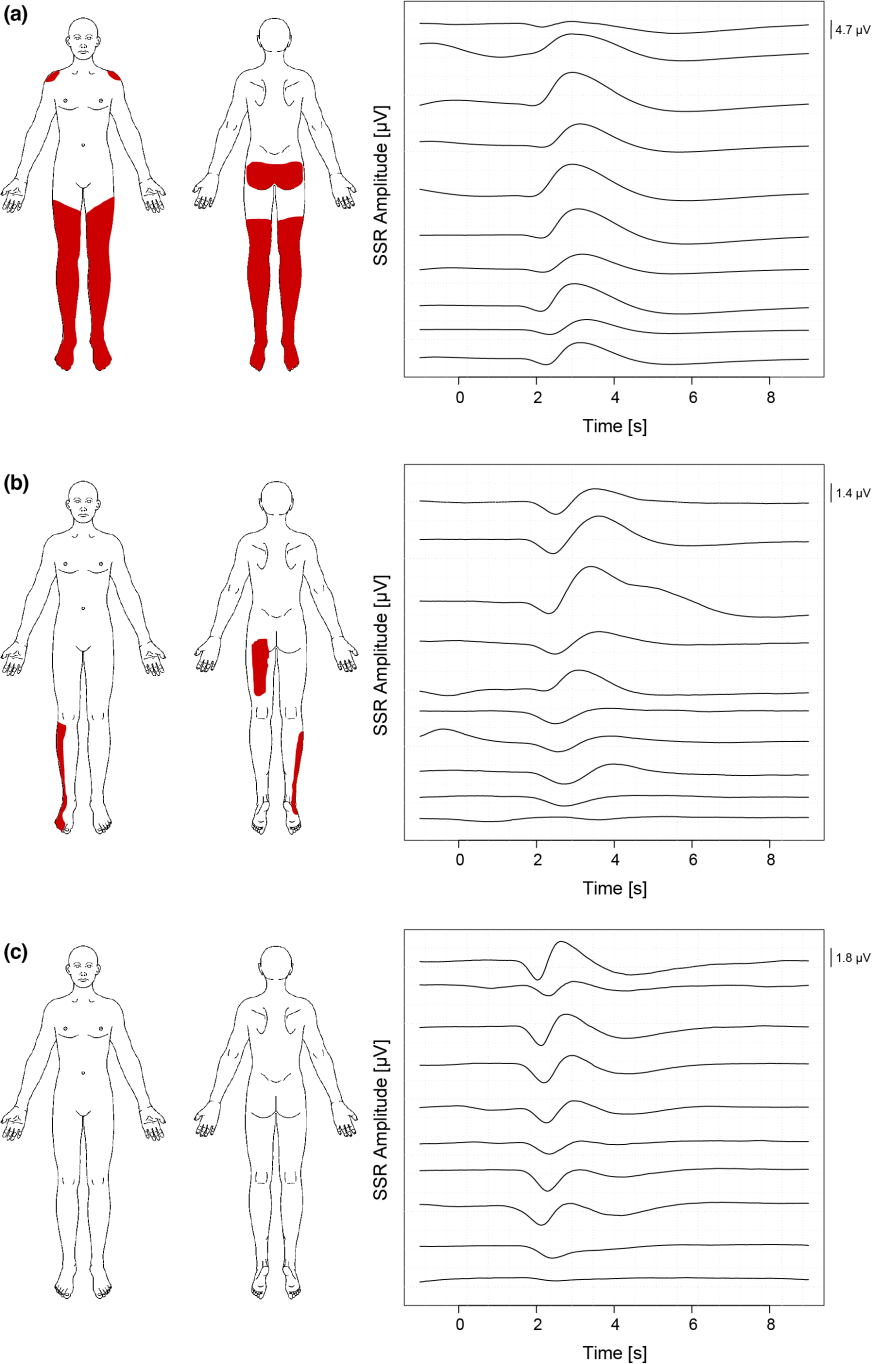
Representative examples of SSR (sympathetic skin response) habituation and neuropathic pain extent. (a) SCI subject with 34.2% of the total body area affected by spontaneous neuropathic pain and a deficient SSR habituation (+7.3% compared to baseline). (b) SCI subject with 4.9% neuropathic pain extent and a pronounced SSR habituation (−59.7% compared to baseline). (c) Healthy control with a pronounced SSR habituation (−54.4% compared to baseline). SCI, spinal cord injury; SSR, sympathetic skin response.

Figure 5 shows three representative examples of SSR habituation and NP extent. It illustrates the relationship between SSR habituation and NP extent. Deficient habituation was seen in an SCI subject with high NP extent (34.2% of body surface, Figure 5a), whilst SSR habituation was still preserved and comparable to that seen in a healthy volunteer (Figure 5c) and a subject with less extensive NP distribution (4.9% of body surface, Figure 5b). With regard to evoked pain, no difference in SSR habituation (*p* = 0.865) was found between subjects with and without allodynia (at or below the lesion level). Further, no significant correlation was found between SSR habituation and the NLI (*p* = 0.508, rho = −0.128). This signified that the amount of deafferentation likely does not affect the SSR readout stimulated and recorded above the level of injury.

Regarding the relationship between TSP magnitude and SSR habituation, no significant overall correlation was found (*p* = 0.608, *r* = 0.097). However, on an individual level, we could observe that the three subjects with the highest NP intensity either showed an enhanced TSP magnitude (up to 584%) or a deficient SSR habituation (an increase in SSR amplitude up to 30.9%).

## 4 DISCUSSION

This study presents novel lines of evidence regarding enhanced TSP during tonic heat application in SCI‐NP compared to SCI‐nonNP and HC. Our results highlight the significance of a prolonged heat application to track the full temporal spectrum of changes in pain modulatory processing. Additionally, our results reveal a relationship between NP extent and altered pain‐autonomic interaction, i.e. reduced habituation of heat‐induced SSR. In summary, this study supports the value of surrogate markers for central sensitization, i.e. enhanced TSP and reduced SSR habituation, in a chronic SCI cohort mainly suffering from widespread spontaneous NP.

### 4.1 Increased temporal summation of pain in neuropathic pain after spinal cord injury

Our results of increased TSP magnitude are in line with studies in chronic SCI‐NP (Defrin et al., 2001; Eide et al., 1996; Gruener et al., 2016; Konopka et al., 2012) as well as other chronic pain syndromes, e.g. fibromyalgia (Price et al., 2002; Staud et al., 2001; Staud et al., 2008), pain after a whiplash injury (Curatolo et al., 2001) and cancer‐related pain (Edwards et al., 2013). Overall, general hypersensitivity is indicated by increased TSP or wind‐up pain in response to a variety of sensory stimuli (cold, heat, mechanical and electrical). Studies in SCI mainly reported increased wind‐up pain in SCI‐NP compared to SCI‐nonNP and HC when repetitively applying von Frey hairs or weighted pinprick devices within painful body regions (Defrin et al., 2001; Eide et al., 1996; Konopka et al., 2012). Whilst these tests are mainly targeting segmental spinal mechanisms (Vogel et al., 2017), further investigations on potential widespread spinal and supraspinal mechanisms involved in central sensitization are warranted. This might be achieved via additional testing of sensory intact sites above the lesion level (Arendt‐Nielsen et al., 2018). TSP enhancement found remote from the distribution of NP symptoms provides indirect support for increased nociceptive sensitivity above the lesion level. The hyperexcitability of residual nociceptive neurons is assumed to spread cranially, e.g. cervical spinal cord, thalamus and cortical regions. There is ample preclinical evidence supporting these mechanisms, revealing neuroinflammation and astrocytic activation in above‐level sites in SCI (Carlton et al., 2009; Nesic et al., 2005). In human studies, above‐level changes have been mainly assessed with magnetic resonance imaging and psychophysical readouts (Ducreux et al., 2006; Gustin et al., 2010; Gustin et al., 2014; Huynh et al., 2021; Murray et al., 2010; Wrigley et al., 2009). Gruener et al. (2016) applied a tonic (30‐s) hot water bath with the immersion of the whole hand and reported enhanced TSP in SCI‐NP in agreement with our findings, being related to generalized hyperexcitability (Gruener et al., 2016). In contrast, Albu et al. (2015) could not observe TSP in any group (SCI‐NP, SCI‐nonNP, HC) which is possibly attributable to their test design. In their study, they applied a contact heat stimulus to a focal skin area (thermode: 572.5 mm^2^) and only for 30 s which mainly led to pain habituation but no TSP (Albu et al., 2015). Interestingly, our results on the nociceptive processing resulted in no group differences at 30, 60 and 90 s, whereas the TSP magnitude reflected significantly increased values in SCI‐NP at 120 s. In conclusion, this highlights the benefit of prolonged test stimulus application whilst investigating TSP.

Based on findings in other chronic pain conditions (Kutch et al., 2017; Willett et al., 2020), we hypothesized that surrogate markers of central sensitization will correlate with NP characteristics. However, our results regarding a relationship of TSP magnitude with NP extent or intensity could not confirm this hypothesis. In contrast, Gruener et al. (2016) reported that enhanced TSP is associated with NP intensity but not extent (Gruener et al., 2016). Whilst the absence of a significant correlation between TSP magnitude and NP extent in our study is in agreement with their findings, we could not substantiate a relationship with NP intensity.

Another relevant factor in central sensitization is the cognitive‐emotional aspects of pain processing, e.g. magnification and rumination, which have been determined a relevant factor in central sensitization (Brosschot, 2002; Smart et al., 2012). Our results are in line with these findings, indicating a positive correlation between TSP magnitude and PCS score. Previous studies reported increased TSP (Edwards et al., 2006) and pronounced painful aftersensations (Edwards et al., 2013; Woolf, 2011) in subjects with enhanced PCS scores, being discussed as indices of central sensitization.

Although tonic heat application enabled us to study TSP, the mechanism of pain adaptation dominated the first phase of stimulation. Whilst all groups showed a pronounced adaptation of pain ratings largely within the first 20 s of noxious stimulation, previous studies reported a deficient pain adaptation in SCI‐NP and SCI‐nonNP compared to HC in areas above the lesion level (Albu et al., 2015; Gruener et al., 2016). These studies attributed their findings on pain adaptation to a generalized dysfunction in endogenous pain inhibition in the SCI cohort, which can, however, be challenged by the fact that pain adaptation in an early phase of tonic heat application primarily reflects peripheral receptor fatigue (Greffrath et al., 2007; Weissman‐Fogel et al., 2015).

### 4.2 Deficient habituation of sympathetic skin responses in widespread neuropathic pain

Pain‐autonomic markers are assumed to represent a surrogate readout of central sensitization in experimentally‐induced secondary hyperalgesia in HC (Scheuren et al., 2020) as well as in chronic central pain in Parkinson's disease (Schestatsky et al., 2007). In our study, no group difference in SSR habituation between SCI‐NP, SCI‐nonNP and HC was found. This finding is in disagreement with studies on migraine (Ozkul & Ay, 2007) and Parkinson's disease (Schestatsky et al., 2007) where pain patients showed deficient SSR habituation compared to pain‐free cohorts. A possible explanation for this discrepancy is the fact that the subjects in these two studies were off medication, whilst many SCI‐NP subjects in our cohort were under the influence of pain medication. Thus, the medication effect might potentially attenuate nociceptive hyperexcitability and thereby restore SSR habituation. Nevertheless, we showed an association of NP extent with SSR habituation when investigating the SCI‐NP group. Such an association between pain distribution and central sensitization has been shown in hip osteoarthritis (Willett et al., 2020) and chronic pelvic pain (Kutch et al., 2017). Overall, these studies discussed their findings depicting increased excitability of widespread spinal and supraspinal centres which is a fundamental feature of central sensitization. Another objective readout of increased excitability within the central nervous system has been employed by Kumru et al. (2012) reporting deficient habituation of contact heat‐evoked potentials in SCI‐NP (Kumru et al., 2012). In addition, studies reporting subjective outcome measures depicted deficient habituation to noxious test stimuli in various pain conditions, e.g. migraine (de Tommaso et al., 2005; Ozkul & Ay, 2007; Valeriani et al., 2003), radiculopathy (Hullemann et al., 2017), low back pain (Vossen et al., 2015), fibromyalgia (de Tommaso et al., 2011; de Tommaso et al., 2017; Smith et al., 2008), Parkinson's disease (Schestatsky et al., 2007) as well as SCI (Kumru et al., 2012).

In general, not only amplitude and habituation can be investigated with SSR recording, but also their latencies. Our finding of prolonged SSR latencies in the overall SCI cohort is in line with reports of longer SSR latencies in fibromyalgia patients for which an abnormal pain processing at the central level was considered, suggesting a sympathetic system vegetative dysfunction (de Tommaso et al., 2017; Ulas et al., 2006). In SCI, however, we assume that prolonged SSR latencies for supralesional stimulation are more likely a consequence of deafferentation and associated atrophic and microstructural changes in areas above the lesion level (Freund et al., 2012; Freund et al., 2013). Deficient SSR habituation in subjects with severe NP might, on the one hand, result from increased nociceptive responsiveness reflecting changes in the modulation of nociceptive inputs (Vossen et al., 2015). On the other hand, animal studies highlight the impact of decreased anti‐nociceptive mechanisms (Carlton et al., 2009), i.e. spinal disinhibition (Gwak et al., 2006; Zeilhofer, 2008) and loss of descending inhibition (Drake et al., 2021). In human research, the latter has been associated with the spatial extent of SCI‐NP (Gruener et al., 2016). The putative coexistence of pro‐ and anti‐nociceptive mechanisms (Yarnitsky et al., 2014) hampers a more rigorous disentanglement of their specific contribution to the emergence and maintenance of NP within our study design.

The fact that enhanced TSP and deficient SSR habituation were not directly related to each other implies that they are complementary rather than congruent signs of central sensitization. Whilst enhanced TSP resembles a pro‐nociceptive process mainly occurring at the spinal dorsal horn (Woolf & Salter, 2000), deficient heat‐induced SSR habituation relies on anti‐nociceptive processes affecting central autonomic networks (Bingel et al., 2007). Therefore, both measures might play a crucial role in the assessment of increased excitability within the central nervous system in SCI‐NP.

### 4.3 Limitations

This study comes with several limitations. The application of a fixed temperature (45°C) for the tonic heat protocol goes along with varying pain ratings at the end of the heating ramp which is a potential confounder of the TSP magnitude (Vierck et al., 1997; Weissman‐Fogel et al., 2015). In order to address this confounder, the pain rating at the ramp was statistically accounted for. More importantly, the fixed temperature did not evoke any pain sensation during the 2‐min heat application in 11 subjects. This likely would have benefited from using individually adjusted temperatures for tonic heat application, however, was limited due to safety restrictions of the device. Further, whilst the medication intake was stable for months, the subjects were not taken off pain medication for the purpose of study participation. In particular, the influence of pain medication reducing central neuronal excitability, e.g. opioids and gabapentinoids, was not controlled for. A major shortcoming in studying pain‐related autonomic measures as estimates for sensitization comes with the fact that the pain and autonomic nervous system are closely linked both neuroanatomically and functionally. Therefore, the assumed hyperexcitability in the nociceptive and/or autonomic pathways cannot be disentangled and future studies are warranted.

## 5 CONCLUSION

In conclusion, our findings indicated a potentially increased excitability of nociceptive and autonomic pathways above the lesion level is present particularly in subjects with widespread chronic NP after SCI. This study further supports the incorporation of tonic heat and simple, objective pain‐autonomic readouts as surrogate markers of central sensitization in NP conditions. A potential implication for clinical practice is constituted by an improved assessment of increased nociceptive sensitivity at the individual level, potentially assisting the prediction of expected benefits from pharmacological treatment of neuronal hyperexcitability based on mechanistic insights. More precise disentanglement of pro‐ and anti‐nociceptive mechanisms underlying chronic pain conditions warrants future mechanistic studies.

## FUNDING INFORMATION

This research was supported by the University of Zurich–Clinical Research Priority Program (Pain), the Swiss National Science Foundation (320030_169250) and the Swiss Spinal Cord Injury Cohort Study (2016‐N‐005, JR).

## CONFLICT OF INTEREST

The authors declare that there are no conflicts of interest regarding this work.
